# Editorial: Molecular biomarkers in the prediction, diagnosis, and prognosis of neurodegenerative diseases

**DOI:** 10.3389/fnins.2023.1226675

**Published:** 2023-06-08

**Authors:** Yuzhen Xu, Shenfeng Qiu, Wenjun Tu, Jun Xu

**Affiliations:** ^1^Department of Rehabilitation, The Second Affiliated Hospital of Shandong First Medical University, Taian, China; ^2^Basic Medical Sciences, University of Arizona College of Medicine-Phoenix, Phoenix, AZ, United States; ^3^Peking Union Medical College, Chinese Academy of Medical Sciences, Beijing, China; ^4^Department of Neurology, China National Clinical Research Center for Neurological Diseases, Beijing Tiantan Hospital, Capital Medical University, Beijing, China

**Keywords:** molecular mechanism, biomarkers, prediction, diagnosis, prognosis, neurodegenerative diseases

Neurodegenerative diseases including Alzheimer's disease (AD), Parkinson's disease (PD), stroke, mental illness and amyotrophic lateral sclerosis (ALS) are characterized by the progressive loss of structure and function of neurons in the central nervous system and has posed a significant burden on individuals, families, and societies worldwide. There has been a growing interest in the identification and utilization of molecular biomarkers as valuable tools in the prediction, diagnosis, and prognosis of neurodegenerative diseases. Molecular biomarkers encompass various biological molecules, such as proteins, nucleic acids, lipids, metabolites, and extracellular vesicles, which can be measured in biological samples, including blood, cerebrospinal fluid (CSF), and even urine. These biomarkers exhibit specific alterations in their expression patterns or structural characteristics that are associated with the underlying neurodegenerative processes (Hansson, 2021). In addition, the emergence of advanced technologies, such as genomics, proteomics, metabolomics, and high-throughput screening platforms, has enabled the identification and validation of numerous molecular biomarkers with potential clinical applications. In recent years, the field of molecular biomarkers has emerged as a promising avenue for enhancing the prediction, diagnosis, and prognosis of neurodegenerative diseases. This topic explores the potential of molecular biomarkers and their transformative role in improving patient outcomes.

AD is usually a slow onset, progressive neurodegenerative disease that accounts for about 70% of all dementias (Zvěrová, 2019). The main reason for the significant difference in lifespan of AD patients may be due to the presence of genetic and clinical heterogeneity in AD, which deserves further studies. To investigate the potential drivers, Zhang et al. identified differentially expressed genes (DEGs) that affect the lifespan of AD patients by bioinformatics analysis. The results suggest genes that exhibit parallel regulatory orientations in cancer, inflammation and AD, and their use as promising targets may help in developing prevention and treatment strategies in further studies. Zhou Y. et al. summarized the pathogenic mechanism of hippocampal neuroregeneration in AD, which will help to further identify the targets regulating neurogenesis and provide new promising therapeutic approaches. Gong et al. described is a rapid, low-cost and simple method for the diagnosis of AD, namely liquid biopsy, which is maturing and improving. In addition, Gao et al. found in a resting-state functional MRI (fMRI) study that abnormal regional homogeneity in the right caudate nucleus is a potential neuroimaging biomarker for the diagnosis of mild cognitive impairment. Interestingly, the study by Bai et al. found a negative correlation between human ocular atrial aqueous (AH) neurofilament light chain and MMSE, suggesting that AH could play a role in screening individuals at high risk for AD.

PD is the second most common neurodegenerative disease after AD. A study by Tong et al. found that decreased serum Glial cell line-derived neurotrophic factor (GDNF) concentrations in PD patients were associated with impaired executive function, and that a complex synergistic mechanism between GDNF and HVA may partially explain the executive dysfunction in PD patients. Overactive bladder (OAB) symptoms are a common complication in patients with PD affecting the quality of survival. Hou et al. investigated the relationship between OAB and prefrontal cortex (PFC) activation patterns and networks in PD patients using fMRI, suggesting that OAB is associated with decreased PFC function, particularly with overactivation of the left dorsolateral prefrontal cortex (DLPFC). Weng et al. used bioinformatics to predict the substrates of SIRT4 in PD, and the results showed that the peroxisome proliferator-activated receptor (PPAR) signaling pathway is one of its most promising targets, offering new possibilities for drug development in PD. Similarly, Hu et al. used bioinformatics to predict crosstalk gene biomarkers for PD and periodontitis, identifying five potential crosstalk genes namely FMNL1, MANSC1, PLAUR, RNASE6 and TCIRG1, suggesting immunological involvement in the association of the two diseases.

The role of exosomes and miRNAs in neurodegenerative diseases is receiving more attention (Juzwik et al., 2019). Cheng et al. identified more differential miRNAs in the microarrays of amyotrophic lateral sclerosis (ALS) patients and finally determined that hsa-miR-34a-3p, hsa-miR-1306-3p, hsa-miR-199a-3p, and hsa-miR-30b-5p were significantly different in both SOD1-mutated ALS and C9orf72-mutated ALS, suggesting their possible involvement in the mechanism of ALS pathogenesis. Although it is still controversial whether stroke is a neurodegenerative disease, it is generally accepted that stroke can cause secondary neurodegenerative lesions. In a Meta-analysis, Zhao et al. showed that serum bilirubin levels correlated with stroke severity and there were gender differences, but the current study did not clarify the causal relationship between bilirubin and stroke prognosis and further studies are still warranted. A prospective cohort study by Wang et al. found elevated levels of serum Nuclear factor erythroid 2-related factor 2 (Nrf2) in patients after cerebral hemorrhage, suggesting that Nrf2 could be used as a biomarker to predict the prognosis of cerebral hemorrhage.

Neurodegeneration is considered to be one of the etiologies of mental disorders (Wingo et al., 2022). Liu et al. found that Mindfulness-based cognitive therapy (MBCT) improved mood and facial emotion recognition in patients with late-life depression (LLD), with the left superior temporal gyrus (L-STG) playing an important role in this process. And Zhou H. et al. found that high homocysteine levels and white matter abnormalities were all involved in the development of cognitive impairment in patients with LLD. Cognitive impairment is also present in schizophrenic patients. A study by Yang et al. showed that forkhead box P2 gene polymorphism, BMI and gender are factors that influence cognitive impairment in schizophrenia, suggesting that hermaphroditism and FOXP2 rs10447760 may be involved in the effect of BMI on cognitive deficits in schizophrenic patients.

In conclusion, the integration of molecular biomarkers into clinical practice has the potential to revolutionize the field of neurodegenerative disease management. By enabling early detection, accurate diagnosis, and reliable prognostic information, these biomarkers have the capacity to facilitate personalized therapeutic interventions, improve patient outcomes, and advance our understanding of these complex and devastating disorders.

## Author contributions

YX drafted the manuscript. All authors contributed to the article and approved the submitted version.
